# Synergistic effect of farnesyl transferase inhibitor lonafarnib combined with chemotherapeutic agents against the growth of hepatocellular carcinoma cells

**DOI:** 10.18632/oncotarget.22086

**Published:** 2017-10-26

**Authors:** Jialiang Wang, Yifan Lian, Yurong Gu, Hongbo Wang, Lin Gu, Yanlin Huang, Liang Zhou, Yuehua Huang

**Affiliations:** ^1^ Guangdong Provincial Key Laboratory of Liver Disease Research, The Third Affiliated Hospital of Sun Yat-sen University, Guangzhou, China; ^2^ Department of Infectious Diseases, The Third Affiliated Hospital of Sun Yat-sen University, Guangzhou, China

**Keywords:** lonafarnib, hepatocellular carcinoma, synergistic effect, chemoresistance

## Abstract

Hepatocellular carcinoma (HCC) is a common and deadly cancer worldwide and is often refractory to chemotherapy due to the development of multidrug resistance. Lonafarnib is an orally active and potent non-peptidomimetic inhibitor of farnesyl transferase. Here, using *in vitro* HCC cell models, we demonstrated that lonafarnib inhibited tumor proliferation and reduced the activity of mitogen-activated protein kinases pathways. In addition, lonafarnib caused G1 to S phase arrest through the downregulation of Cyclin D1, CDK6 and SKP2, while it induced cellular apoptosis by promoting the cleavage and activation of Caspase-3 and PARP. When combined with doxorubicin and sorafenib, lonafarnib was able to increase the sensitivity of HCC cells to chemotherapy. Furthermore, we also constructed ABCB1-overexpressing HCC cells and found that lonafarnib decreased chemoresistance by inhibiting ABCB1-mediated drug efflux activity. These results suggest that lonafarnib may be a promising synergistic agent for improving the treatment of drug-resistant HCC.

## INTRODUCTION

Liver cancer is the second most common cause of death from cancer worldwide, estimated to account for approximately 700,000 deaths in 2012 [1]. Among the histologically different hepatic neoplasms, hepatocellular carcinoma (HCC) is the most common type of liver cancer with an incidence rate of up to 90% [2]. The prevalence of HCC in China is high, and in particular, 55% of all HCC cases worldwide is reported among the Chinese population [3, 4]. HBV infection is the largest risk factor of HCC in China, while other risk factors include HCV infection, aflatoxin exposure, alcohol consumption and tobacco smoking [5, 6]. Despite remarkable progress in prevention, detection and treatment of cancer over the last five decades, curative treatments including liver transplantation and hepatic resection are only suitable for fewer than 20% of HCC patients and there is no adequate and effective therapy for HCC due to diagnosis at late stage or frequent relapse [7, 8]. Therefore, as a palliative treatment, chemotherapy is routinely used for unresectable HCC patients.

However, HCC is refractory to chemotherapy due to its tendency to develop multidrug resistance (MDR). Most of the currently used cytotoxic drugs, either as single agent or as part of a multidrug regimen, for the treatment of HCC remain unsatisfactory because of the lack of benefit to overall survival (OS) of patients [9]. Sorafenib, a multikinase inhibitor that targets Raf family kinases and several other angiogenesis-related receptor tyrosine kinases (RTKs), is the first approved targeted drug to be used for the treatment of advanced HCC. However, its efficacy is moderate with only a three-month improvement in OS compared to placebo, and acquired resistance often occurs within 6 months [10]. Although several types of mechanisms are related to chemoresistance, such as changes in drug kinetics, amplification of drug targets or plasticity of the tumor microenvironment [11, 12], the one most commonly observed is the abnormal expression of energy-dependent efflux pumps encoded by the ABCB1 gene (P-glycoprotein 1, P-gp or MDR1) or other multidrug resistance-associated proteins (MRPs) [13, 14]. Cytotoxic drugs are rapidly removed from cells through efflux pumps; thus, the intracellular drug concentration becomes lower, and cancer cells acquire resistance to chemotherapeutics.

Efforts have been made to overcome the MDR phenotype and enhance chemosensitivity of HCC cells. First, selective inhibition of ABCB1 expression has been used to reverse drug resistance. ABCB1 antisense RNA has been reported to increase the sensitivity of SMMC7721/ADM cells to anticancer drugs [15]. In addition, many compounds have been shown to diminish drug resistance when coadministered with chemotherapeutic agents. Kim et al. first described that a combination of tamoxifen and cyclosporin A showed significant synergism on the sensitivity to doxorubicin of both low and high ABCB1-expressing HCC cell lines [16]. Recently, Wu et al. also reported that metformin, by silencing NF-κB signaling, could effectively reverse MDR in HCC cells by the downregulation of ABCB1 expression [17]. Even though these strategies show some positive effects on MDR reversal, the innate toxicities of these compounds, for example, off-target effects from antisense RNA constructs, and severe side effects caused by these compounds must be carefully considered [18]. Thus, less toxic and more potent agents are urgently needed for overcoming chemoresistance in cancer therapy.

Lonafarnib is an orally active, potent and selective inhibitor of human farnesyl transferase [19]. Although it was originally developed for cancers associated with Ras signaling whose activation requires farnesylation, anti-proliferative effects of this compound have also been observed on a variety of human tumor cell lines lacking Ras activity including lung, pancreas, colon, prostate, urinary bladder and hematological cancer, both *in vitro* and *in vivo* [20–22]. However, as a single agent, its activity among patients with solid tumors was weak in clinical trials. There is emerging interest in lonafarnib as an additive or synergistic drug with various cytotoxic and targeted agents [23–25]. Single-agent lonafarnib was able to reverse imatinib resistance in chronic myeloid leukemia [26]. Enhanced antitumor activity has also been reported in preclinical cancer models of lung cancer when lonafarnib was combined with 5-fluorouracil or taxanes [27]. In addition, low concentration of lonafarnib exhibits significant suppression of ABCB1 activity in NIH-G185 cells when coadministered with ABCB1 substrates or inhibitors [28]. Therefore, lonafarnib holds potential as a coadministered agent to overcome chemoresistance in HCC.

In this study, we investigated the effect of lonafarnib alone or in combination with chemotherapeutics on two HCC cell lines, SMMC-7721 and QGY-7703. To validate whether lonafarnib contributes to the reversal of the MDR phenotype in HCC, we also constructed HCC cell models stably overexpressing ABCB1. Treatment of ABCB1-overexpressing cells with lonafarnib showed positive results on growth inhibition of chemoresistant HCC cells through ABCB1-mediated mechanism. Our study provided *in vitro* evidence in supporting the synergistic usage of lonafarnib for the treatment of HCC and in particular, in decreasing chemoresistance to commonly used chemotherapeutics in HCC.

## RESULTS

### Lonafarnib inhibits growth of human HCC cells

**Figure 1:**
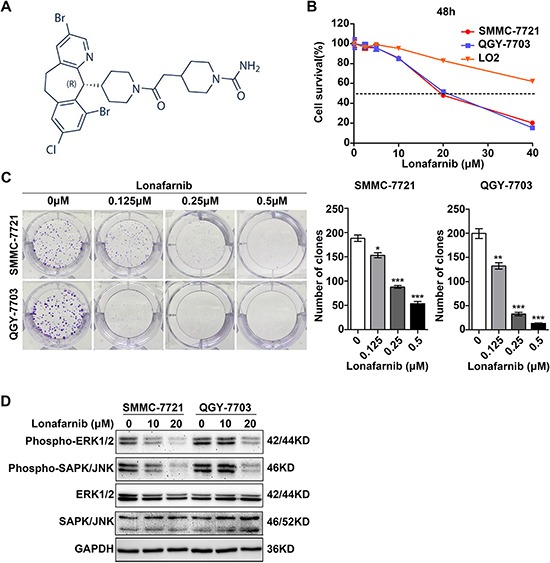
Lonafarnib inhibits growth of human HCC cells (**A**) Chemical structure of lonafarnib. (**B**) Dose escalation effect of lonafarnib on cell viability. HCC cell lines (SMMC-7721 and QGY-7703) and the immortalized hepatic cell line LO2 were incubated with different concentrations of lonafarnib as indicated; the IC50 value at 48 h was determined in these cell lines: SMMC-7721 (20.29 μM), QGY-7703 (20.35 μM) and LO2 (undetectable). CCK-8 assay was used to detect the cell viability and the dashed line indicated the IC50 line. The data are presented as the means ± SD. (**C**) Colony formation assay in SMMC-7721 and QGY-7703 cells. After lonafarnib treatment, cells were fixed and stained with crystal violet. Left panel: representative image of colonies; right panel: the number of colonies is summarized and presented as the mean ± SD. ^*^P < 0.05; ^**^P < 0.01; ^***^P < 0.001. (**D**) Western blot analysis of protein levels of phospho-ERK1/2, phospho-SAPK/JNK, total ERK1/2 and total SAPK/JNK in HCC cells treated with lonafarnib as indicated.

The chemical structure of lonafarnib was shown in Figure 1A [21]. To investigate the cytotoxicity of lonafarnib, we performed CCK-8 assay to measure cell viability after treatment with various drug concentrations for different time points in two representative HCC cell lines, SMMC-7721 and QGY-7703, and an immortalized liver cell line LO2. Lonafarnib markedly suppressed the proliferation of the HCC cell lines SMMC-7721 and QGY-7703 in a dose-dependent manner. The IC50 values at 48 h for these two HCC cell lines were 20.29 μM and 20.35 μM, respectively. However, lonafarnib exerted limited growth inhibition toward the hepatic cell line LO2 (Figure 1B). In addition, colony formation significantly decreased after lonafarnib treatment with lonafarnib in SMMC-7721 and QGY-7703 cells (Figure 1C). Furthermore, Western blotting showed an obvious reduction in protein levels of phospho-ERK1/2 and phospho-SAPK/JNK in the HCC cell lines (Figure 1D). These results suggest that lonafarnib inhibits growth of human HCC cells but only has limited effect on the viability of hepatic cells.

### Lonafarnib induces apoptosis in HCC cells

**Figure 2:**
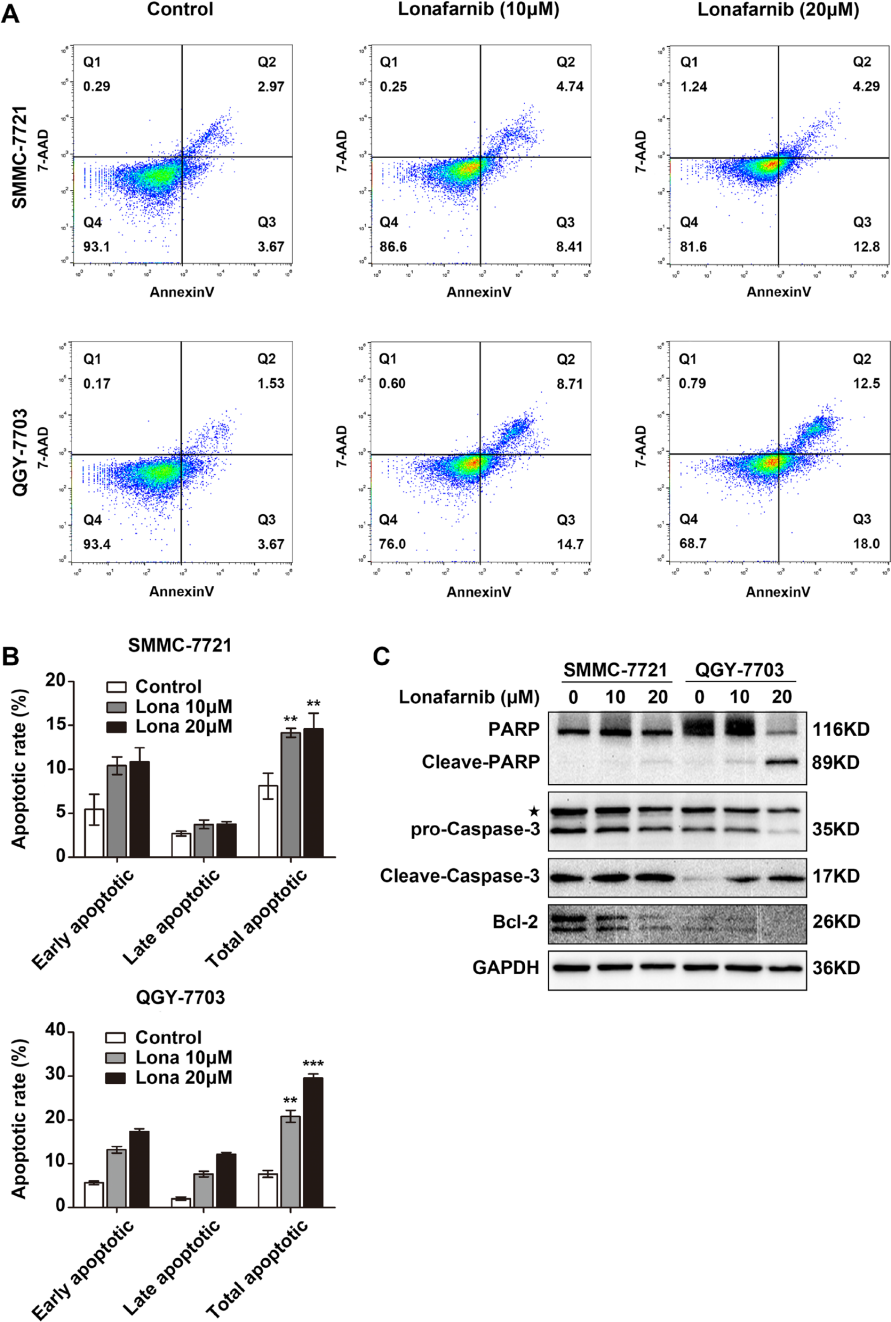
Lonafarnib induces apoptosis in HCC cells (**A**) Representative image of flow cytometric analysis of apoptotic SMMC-7721 and QGY-7703 cells stained with Annexin V-FITC/7-AAD after lonafarnib treatment. (**B**) The flow cytometry results are summarized in the panel. The data are presented as the means ± SD. ^**^P < 0.01; ^***^P < 0.001. (**C**) Western blot analysis of levels of apoptosis-related protein including PARP, cleaved PARP, pro-Caspase-3, cleaved Caspase-3 and Bcl-2 in HCC cells treated with lonafarnib as indicated. The asterisk indicates a non-specific band.

Chemotherapy is often associated with cellular apoptosis. To determine whether lonafarnib also induces apoptosis, we stained HCC cells with Annexin V-PE and 7-AAD after treatment. The percentage of total apoptotic cells increased in a dose-dependent manner, and in the 20 μM-treated group of SMMC-7721 and QGY-7703 cells, the percentage increased by 2- or 3-fold compared to that of the corresponding control groups (Figure 2A and 2B). Western blotting also confirmed the cleavage and activation of caspase-3 and PARP, two apoptotic markers, and the reduced expression of Bcl-2 in the lonafarnib-treated HCC cells (Figure 2C). These results indicate that lonafarnib can induce apoptosis in HCC cells.

### Lonafarnib causes G1 to S phase arrest in HCC cells

**Figure 3:**
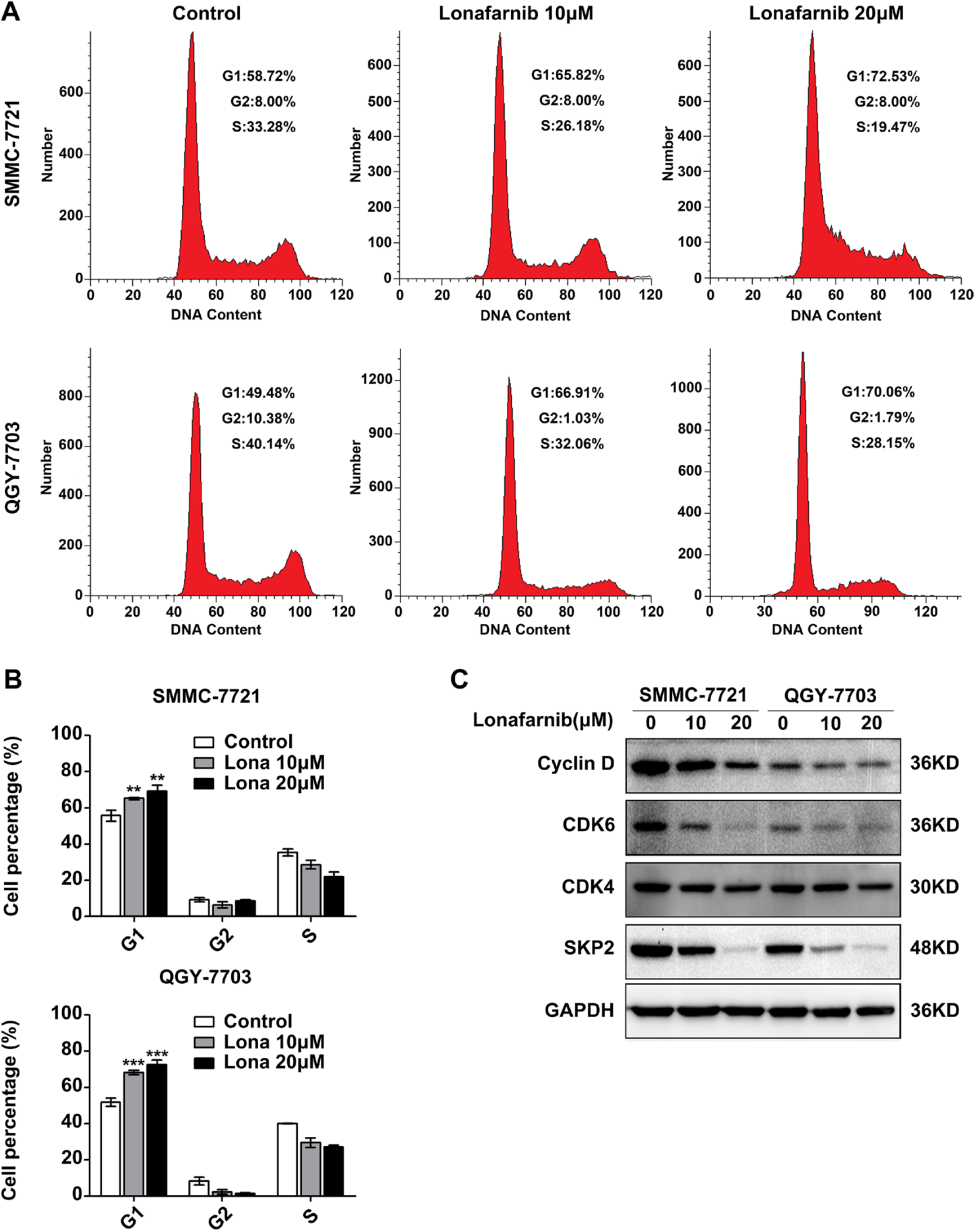
Lonafarnib causes G1 to S phase arrest in HCC cells (**A**) Representative image of flow cytometric analysis of cell cycle distribution of SMMC-7721 and QGY-7703 cells after lonafarnib treatment as indicated. (**B**) The flow cytometry results are summarized in the panel. The data are presented as the means ± SD. ^**^P < 0.01; ^***^P < 0.001. (**C**) Western blot analysis of levels of cell cycle-related proteins including Cyclin D, CDK4, CDK6 and SKP2 in HCC cells treated with lonafarnib as indicated.

To further clarify the mechanism related to the growth suppression of HCC cells after lonafarnib treatment, we analyzed cell cycle distribution using flow cytometry. We found that the percentage of HCC cells in the G1 phase significantly increased after lonafarnib treatment compared to that in the control group (Figure 3A and 3B). Consistent to these data, the expression level of Cyclin D1, CDK6 and SKP2 but not of CDK4, which are all important proteins required for G1 to S phase transition, decreased after treatment (Figure 3C). These results suggest that lonafarnib causes G1 to S phase arrest in HCC cells.

### Lonafarnib displays synergistic effect with doxorubicin and sorafenib on HCC cells

**Figure 4:**
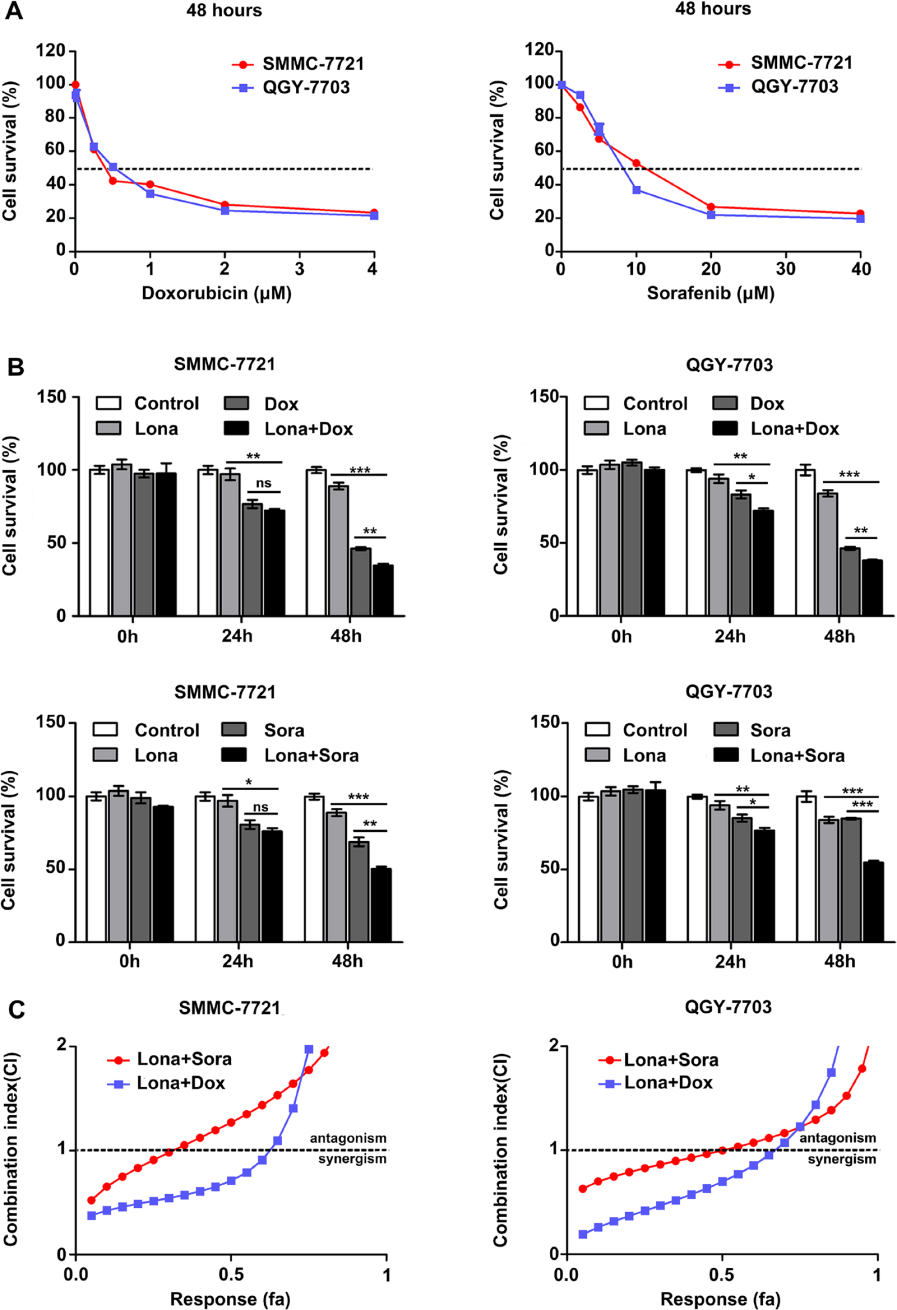
Lonafarnib displays a synergistic effect with doxorubicin and sorafenib (**A**) Dose escalation effect of doxorubicin and sorafenib on the viability of HCC cells measured by CCK-8 assay. The dashed line indicated the IC50 line and the IC50 value for both drugs at 48 h was determined in these cell lines. SMMC-7721: doxorubicin (0.25 μM) and sorafenib (10.37 μM); QGY-7703: doxorubicin (0.38 μM) and sorafenib (8.86 μM). The data are presented as the means ± SD. (**B**) CCK-8 assay in HCC cells treated with low and appropriate doses of doxorubicin and sorafenib in combination with 2.5 μM lonafarnib as indicated. Cell viability was examined 48 hours after treatment using the CCK-8 assay. The data are presented as the means ± SD. NS, not significant; ^*^P < 0.05; ^**^P < 0.01; ^***^P < 0.001. (**C**) HCC cells were treated using increasing concentrations of lonafarnib and doxorubicin or sorafenib, either alone or at a fixed ratio (1:10 for doxorubicin and 1:1 for sorafenib). The combination index was calculated as described in Materials and Methods and is plotted vs. affected fraction.

Conventional cytotoxic drugs or sorafenib are not that effective as monotherapy in treating HCC. To determine whether lonafarnib can enhance the antitumor effect of chemotherapeutics, we coadministered lonafarnib with doxorubicin and sorafenib in HCC cells. The dose-response curves of these chemo agents are shown in Figure 4A. The low and appropriate concentrations of sorafenib (2.5 μM) or doxorubicin (0.25 μM) were determined and chosen to be combined with 2.5 μM lonafarnib for treating HCC cells. We found that after 48 hours of treatment, compared to the single agent groups (Dox or Sora), the lonafarnib combination groups with doxorubicin or sorafenib (Lona + Dox or Lona + Sora) displayed a robust reduction in cell viability (Figure 4B). To further investigate the concentration range of effective synergism, HCC cells were exposed to lonafarnib, doxorubicin or sorafenib alone or in combination at a fixed ratio, and the affected fraction (fa) values were determined (Table 1). The CI-fa curve showed that under the indicated concentrations (fa < 0.65 for the ‘Lona+Dox’ group and fa < 0.35 for the ‘Lona+Sora’ group in SMMC-7721 cells; fa < 0.55 for the ‘Lona+Dox’ group and fa < 0.70 for the ‘Lona+Sora’ group in QGY-7703 cells), the values of the combination index (CI) were less than 1, confirming the synergistic effect of lonafarnib with doxorubicin or sorafenib (Figure 4C). These results indicate that lonafarnib exerts a synergistic effect with doxorubicin and sorafenib on HCC cells.

**Table 1:** Values of affected fraction (fa) of HCC cells in response to lonafarnib, doxorubicin and sorafenib as single agents or in combination at various concentrations

	Lona (μM)	fa	Dox (μM)	fa	Sora (μM)	fa	Lona + Dox (μM)	fa	Lona + Sora (μM)	fa
SMMC-7721	2.50	0.05	0.25	0.40	2.50	0.07	2.50 + 0.25	0.44	2.50 + 2.50	0.10
	5.00	0.11	0.50	0.56	5.00	0.23	5.00 + 0.50	0.60	5.00 + 5.00	0.43
	10.00	0.18	1.00	0.62	10.00	0.41	10.00 + 1.00	0.66	10.00 + 10.00	0.63
	20.00	0.45	2.00	0.64	20.00	0.67	20.00 + 2.00	0.68	20.00 + 20.00	0.66
	40.00	0.71	4.00	0.66	40.00	0.70	40.00 + 4.00	0.68	40.00 + 40.00	0.64
QGY-7703	2.50	0.26	0.25	0.38	2.50	0.15	2.50 + 0.25	0.47	2.50 + 2.50	0.24
	5.00	0.28	0.50	0.51	5.00	0.30	5.00 + 0.50	0.66	5.00 + 5.00	0.56
	10.00	0.38	1.00	0.69	10.00	0.57	10.00 + 1.00	0.74	10.00 + 10.00	0.80
	20.00	0.62	2.00	0.77	20.00	0.81	20.00 + 2.00	0.75	20.00 + 20.00	0.83
	40.00	0.85	4.00	0.79	40.00	0.85	40.00 + 4.00	0.83	40.00 + 40.00	0.82

### ABCB1 overexpression causes the chemoresistance phenotype in HCC cells

**Figure 5:**
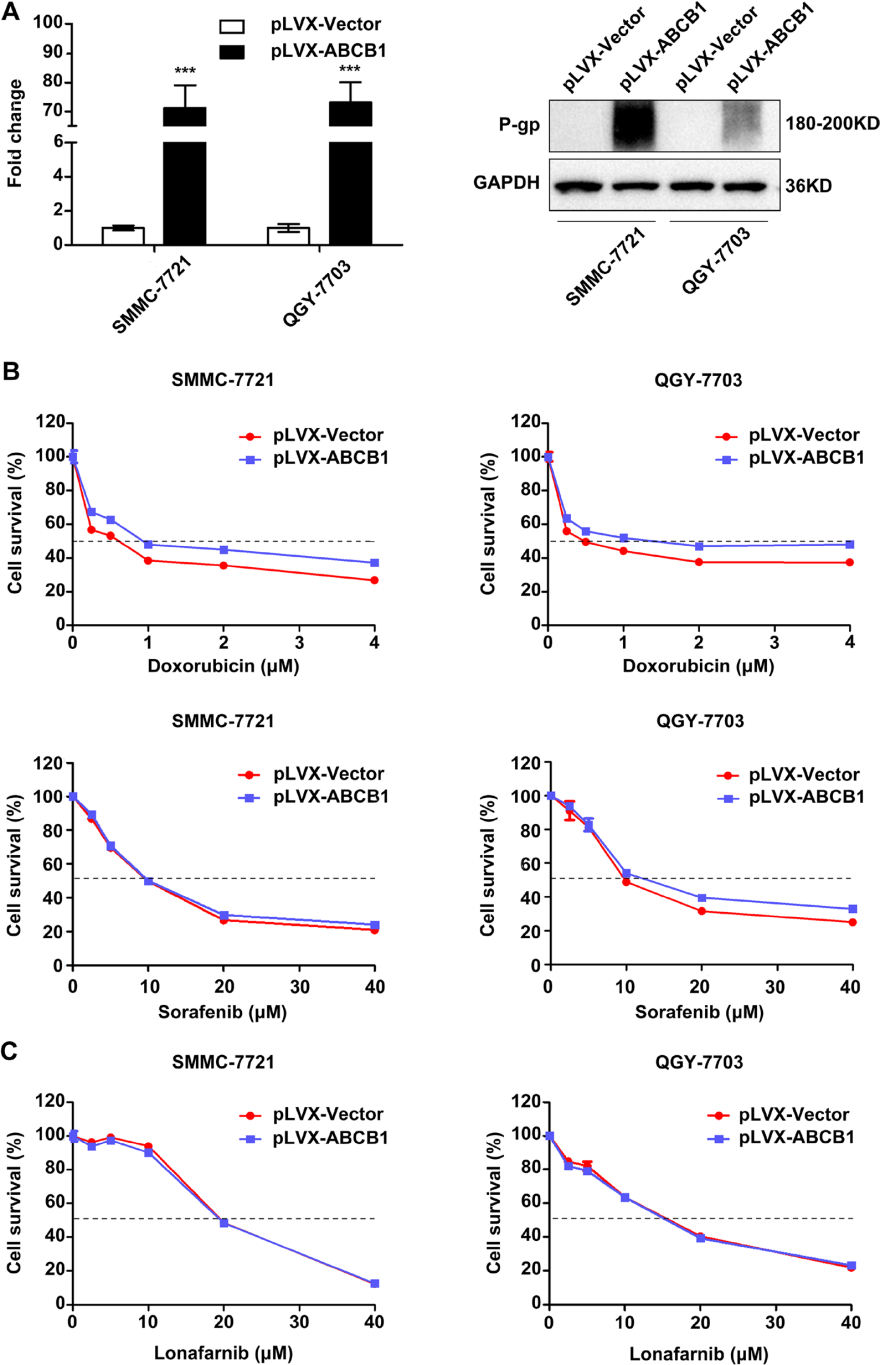
ABCB1 overexpression promotes the chemoresistance phenotype in HCC cells (**A**) The mRNA (left panel) and protein (right panel) expression levels of ABCB1 in lentivirus infected SMMC-7721 and QGY-7703 cells. The data are presented as the means ± SD. ^***^P < 0.001. (**B** and **C**) Cytotoxic effect of doxorubicin, sorafenib (B) and lonafarnib (C) on the viability of control and ABCB1-overexpressed HCC cells. Cells were cultured in the presence of different concentrations of doxorubicin (0, 0.25, 0.5, 1, 2 and 4 μM), sorafenib (0, 2.5, 5, 10, 20 and 40 μM) and lonafarnib (0, 2.5, 5, 10, 20 and 40 μM) for 48 h, and cell viability was determined using the CCK-8 assay. The data are presented as the means ± SD.

HCC is often associated with multidrug resistance. The mechanism commonly involved in the development of the multidrug resistance phenotype in HCC cells is the abnormal expression of ABCB1. To investigate whether lonafarnib could reverse ABCB1-mediated chemoresistance in HCC, we first constructed stably ABCB1-overexpressing SMMC-7721 and QGY-7703 cells through lentiviral infection (Figure 5A). ABCB1 overexpression indeed rendered the HCC cells more refractory to doxorubicin but not to sorafenib. Sorafenib is known to have the ability to reverse ABCB1-mediated MDR phenotype [29]. ABCB1-overexpressing cells had larger IC50 values for doxorubicin but not for sorafenib compared to the corresponding IC50 values of the control cells (Figure 5B). Interestingly, the sensitivity of ABCB1-overexpressing and control cells to lonafarnib was not different (Figure 5C). These results suggest that ABCB1 overexpression is involved in chemoresistance to doxorubicin in HCC cells but has no effect on sorafenib or lonafarnib treatment.

### Lonafarnib reduces the ABCB1-mediated chemoresistance in HCC cells

**Figure 6:**
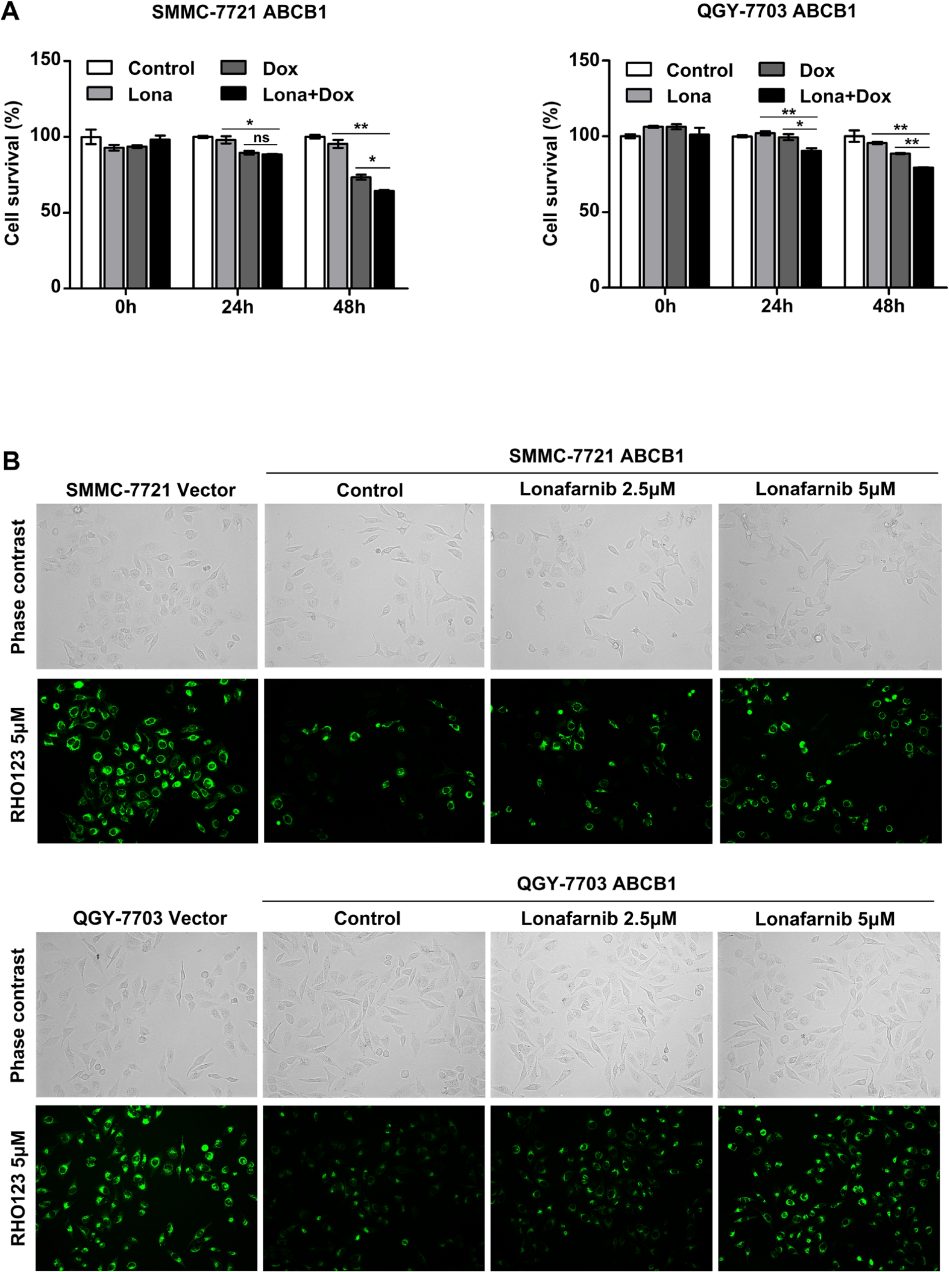
Lonafarnib reduces ABCB1-mediated chemoresistance in HCC cells (**A**) ABCB1-overexpressing HCC cells were treated with 0.25 μM doxorubicin in combination with 2.5 μM lonafarnib, and cell viability was determined 48 h after treatment using the CCK-8 assay. The data are presented as the means ± SD. NS, not significant; ^*^P < 0.05; ^**^P < 0.01. (**B**) RHO123 staining assay of ABCB1-overexpressing HCC cells. Cells were stained with 5 μM RHO123 after lonafarnib treatment as indicated. Intracellular fluorescence was observed and photographed under a microscope to measure cellular efflux pump activity.

Since lonafarnib was not sensitive to ABCB1 overexpression and a previous report stated that lonafarnib could suppress the activity of ABCB1 [28], we asked whether lonafarnib was effective in reversing the ABCB1-mediated chemoresistance in HCC cells. We found that after 48 hours of coadministration, low dose of lonafarnib (2.5 μM) significantly reduced the viability of ABCB1-overexpressing cells compared to that of the cells treated with doxorubicin alone (Figure 6A). A previous study had reported that the ABCB1-mediated MDR phenotype was related to the ATPase-dependent efflux pump activity of ABCB1 [30]. Through RHO123 staining assay, where the fluorescence staining served as a marker of cellular efflux activity, we further demonstrated an increase in intracellular fluorescence staining of ABCB1-overexpressing cells in a dose-dependent manner in response to lonafarnib treatment (Figure 6B). These results suggest that lonafarnib was able to reduce the ABCB1-mediated chemoresistance in HCC cells mainly by inhibiting its efflux pump activity.

## DISCUSSION

Chemotherapeutic treatment is not routinely used in HCC because of innate or acquired chemoresistance and adverse events. Although clinical trials have been conducted with numerous drugs and treatment methods in HCC, almost all trials have ended in failure and could not demonstrate any significant improvement in patient survival. Currently, sorafenib is the only drug that was shown to have survival benefit for advanced HCC. The lack of secondary therapeutic options is clinically problematic. Only 20% of HCC patients are eligible for radical therapy comprising hepatectomy or liver transplantation, and even if a radical cure is achieved in HCC, the recurrence rate is high owing to the underlying liver diseases [31]. Thus, the majority of HCC patients eventually require systemic chemotherapy, and thus it is urgently needed to discover novel effective drugs for HCC treatment [32, 33].

It is well known that the multistep development of human tumors include ten acquired biological characteristics termed the hallmarks of cancer [34]. Among them, sustaining proliferative signaling, evading growth suppression and resisting cell death are three principal and important aspects, which are also applicable to HCC. In the present study, we first showed that when used as a single agent, lonafarnib was able to inhibit cellular proliferation and colony formation in HCC cells. Since the constitutive signaling of the RAF-MEK-Mitogen Activated Protein Kinase (MAPK) cascade plays an important role in sustaining tumor proliferation, we were also able to demonstrate that treatment with lonafarnib reduced the expression of phospho-ERK1/2 and phospho-SAPK/JNK, two effector molecules of MAPK pathway (Figure 1). In addition, lonafarnib alone was sufficient to induce cellular apoptosis in HCC cells *in vitro*. The cleavage and activation of Caspase-3 and PARP, two primary markers for cellular apoptosis, were detected by Western blotting after lonafarnib treatment (Figure 2). Furthermore, cell cycle dysregulation has been shown to be involved in tumor growth inhibition. Using flow cytometric analysis, we also found that administration of lonafarnib arrested HCC cells in G1 phase possibly due to the reduced expression of Cyclin D1 and CDK6, both of which drive G1/S phase transition and promote cell cycle progression by forming a protein complex (Figure 3). Therefore, our results showed that lonafarnib inhibited cellular proliferation, caused cell cycle arrest and induced apoptosis through various molecular mechanisms, which indicated that lonafarnib alone was effective for *in vitro* treatment of HCC cell models.

The development of chemoresistance is a major obstacle in the successful and effective chemotherapeutic treatment of HCC. HCC is thought to be intrinsically chemorefractory, as it easily acquires chemoresistance [35, 36]. Therefore, in order to increase the sensitivity of HCC to the conventional chemotherapeutics, we combined lonafarnib with two separate chemo agents and found that low dose of lonafarnib significantly enhanced the killing effect of doxorubicin and sorafenib on HCC cells (Figure 4). The mechanism by which HCC acquired chemoresistance is varied. The biochemical pathways could include altered drug influx/efflux or targets, increased drug metabolism, enhanced DNA repair following damage and suppressed apoptotic effector pathways. One of the most important causes of resistance in cancer cells is the expression of the drug transporter family known as ATP-binding cassette (ABC) transporters. The most common member of this family is ABCB1 whose overexpression has been reported to be associated with the reduced intracellular accumulation of doxorubicin and sorafenib in HCC cells and with worse prognosis in patients. Thus, we constructed HCC cell models stably overexpressing ABCB1 and verified that ABCB1 overexpression indeed increased the IC50 value of doxorubicin in HCC cells. Next, we asked whether low dose of lonafarnib was effective in reversing the ABCB1-mediated chemoresistance and demonstrated that treatment combining low dose of lonafarnib with doxorubicin induced substantial cell death mainly through the inhibition of drug efflux activity (Figure 6), suggesting lonafarnib as a potential synergistic agent. These results indicate that lonafarnib could enhance chemosensitivity partly through the ABCB1-mediated pathway in HCC cells.

Lonafarnib, first reported as a potent non-peptidomimetic inhibitor of farnesyl transferase in the 1990s, blocks farnesylation of H-Ras and K-Ras-4B with *in vitro* IC50 values of 1.9 and 5.2 nM, respectively [19]. Since its advent, extensive preclinical and clinical studies have been conducted to confirm its effect on a wide variety of solid and hematological malignancies. Lonafarnib was reported to inhibit the growth of human tumor cell lines in culture and tumor xenografts with or without activated Ras signaling. Its synergistic growth inhibitory effect has been observed with cisplatin, docetaxel, paclitaxel and sorafenib *in vitro* using tumor cell lines. Lonafarnib also exhibited anti-angiogenic effect on non-small cell lung cancer and head and neck squamous carcinoma cells [37]. Lonafarnib was reported to be involved in various molecular pathways, including downregulation of the PI3K/AKT/mTOR pathway and Caspase-dependent apoptosis [38] as well as induction of cell cycle arrest [39]. However, to the best of our knowledge, our study is the first report to demonstrate lonafarnib, alone or in combination with other chemotherapeutics, to be effective in inhibiting the growth of HCC cells *in vitro*, which is consistent with the above studies.

ABCB1 expression level is often elevates in many tumor cells. This active efflux pump renders tumors more chemorefractory so that they require higher doses of drugs, which eventually result in increased toxicity to normal cells. Interestingly, Wang et al. proposed that the inhibition of ABCB1 by lonafarnib may be serendipitous [28]. Other researchers have also suggested that other than Ras protein, the potential molecular targets of lonafarnib could include mitotic proteins CENP-E and CENP-F, small GTPases RhoB and Rheb, and HDJ-2, all of which require farnesylation for their functional activity [40, 41]. Thus, the reversal of chemoresistance by lonafarnib may possibly rely on other cellular proteins besides ABCB1. Further study should be conducted to validate the precise cellular targets of lonafarnib. In addition, the most common adverse events of lonafarnib are fatigue, diarrhea, nausea and anorexia in a dose-dependent manner [22]. Our study showed that other than the limited growth inhibition of the hepatic cell line, low dose of lonafarnib could significantly increase the sensitivity of HCC cells to chemotherapy, which indicates that with a manageable side effect profile, lonafarnib could be a promising synergistic agent to reduce the dosage of chemotherapy in HCC treatment. Since our study only involves *in vitro* cell models, preclinical animal experiments and clinical trials are needed to prove the efficacy of lonafarnib for the treatment of HCC patients in the future.

In summary, our results demonstrate that lonafarnib alone is effective in inhibiting cellular proliferation, causing cell cycle arrest and inducing apoptosis in HCC cells. In addition, lonafarnib displays a significant synergistic effect with other chemotherapeutics and is also able to reduce chemoresistance mediated by the ABCB1 pathway in HCC cells. This study provides compelling evidence in supporting that lonafarnib could be a promising synergistic agent for improving the treatment of drug-resistant HCC.

## MATERIALS AND METHODS

### Reagents

Lonafarnib, sorafenib and doxorubicin were purchased from Selleck Chemicals (TX, USA). Stock solutions of these chemicals were dissolved in dimethyl sulfoxide (DMSO, Sigma-Aldrich) and stored at −80°C. Cell Counting Kit-8 (CCK-8) was purchased from Dojindo Molecular Technologies Inc. (Kumamoto, Japan). Antibodies against Caspase-3 (#9665), cleaved Caspase-3 (#9664), PARP (#9542), Cyclin D (#2978), CDK4 (#12790), CKD6 (#13331), phospho-p44/42 MAPK, (#4370), phospho-SAPK/JNK, (#4668), Bcl-2 (#4223) and SKP2(#2652) were obtained from Cell Signaling Technology (Beverly, USA).

### Cell cultures

The human hepatocellular carcinoma cell lines SMMC-7721 and QGY-7703 and immortalized hepatic cell line LO2 were obtained from the Third Affiliated Hospital of Sun Yat-sen University. All cell lines were thawed from early passage stocks and passaged for less than 6 months. All cell lines were cultured in Dulbecco's Modified Eagle's Medium (DMEM, Life Technologies) supplemented with 10% fetal bovine serum (FBS, Gibco). Cells were grown in a humidified 5% CO_2_ incubator at 37°C and passaged using standard cell culture techniques.

### Stable overexpression of ABCB1 in HCC cells

Full-length human ABCB1 cDNA was amplified from the cDNA library of QGY-7703 cells and subcloned into pLVX-DsRed-Monomer-N1 vector purchased from Clontech. Lentiviruses produced in 293T cells were used to infect SMMC-7721 and QGY-7703 cells by spinfection (500× g for 1.5 h), and the infected cells were incubated overnight, followed by selection with 2 μg/ml of puromycin (P8833, Sigma-Aldrich) for 2 weeks. Stable overexpression of ABCB1 was validated by Western blotting and qPCR.

### Cell viability analysis

The inhibitory effect of lonafarnib on cell viability was assessed with the CCK-8 assay. In total, 3 × 10^3^ cells were seeded in 96-well plates and treated with the different agents as indicated. Cells were either incubated for 24 or 48 hours in the incubator. At the end of these periods, 10 μl CCK-8 reagent was added into each well, and the cells were incubated for another 4 hours. The absorbance (OD value) at 450 nm was then measured using a spectrometer (SpectraMax M5 Microplate Reader, Molecular Devices LLC). IC50 was determined with GraphPad Prism 5.

### Western blotting

Cells were lysed in NETN buffer (20 mM Tris-HCl at pH 8.0, 100 mM NaCl, 1 mM EDTA, 0.5% Nonidet P-40) containing protease and phosphatase inhibitor cocktails (Thermo Fisher, USA). The protein concentration of the lysate was measured using the BCA protein assay kit (Pierce); after normalization, proteins were separated by 8% or 10% SDS-PAGE, transferred to polyvinylidene fluoride (PVDF) membranes and probed with the indicated primary antibodies. The blots were then incubated with species-specific HRP-conjugated secondary antibodies, and the immunoreactive bands were visualized by enhanced chemiluminescence (ECL, Pierce).

### RNA isolation, reverse transcription and real-time quantitative PCR (qRT-PCR)

Total RNA was isolated using the TRIzol reagent according to the manufacturer's instructions (Invitrogen). Subsequently, a total of 2 μg of purified RNA from each sample was reverse transcribed using GoScript^TM^ Reverse Transcription System (Promega). Real-time quantitative PCR was performed with Platinum SYBR Green qPCR SuperMix-UDG (Invitrogen) on a LightCycler 480 PCR platform (Roche). GAPDH mRNA was used as an internal standard reference. Normalized expression was calculated using the comparative C_T_ method, and fold changes were derived from the 2^−ΔΔCt^ values for each gene. The sequences of primers used are as follows: ABCB1 forward: TTGCTGCTTACATTCAGGTTTCA and ABCB1 reverse: AGCCTATCTCCTGTCGCATTA; GAPDH forward: GGAGCGAGATCCCTCCAAAAT and GAPDH reverse: GGCTGTTGTCATACTTCTCATGG.

### Flow cytometry

For apoptosis analysis, cells were stained with Annexin V-PE and 7-AAD (AP104, Multi Sciences) and evaluated by flow cytometry according to the manufacturer's protocol. Briefly, 1 × 10^6^ cells were washed twice with PBS and stained with 5 μl Annexin V-PE and 10 μl 7-AAD in 1× binding buffer for 15 min at room temperature in the dark. Apoptotic cells were determined using a Beckman-Coulter Flow Cytometry FC500. Both early (Annexin V-positive/7-AAD-negative) and late (Annexin V-positive/7-AAD-positive) apoptotic cells were included when assessing cell death.

For cell cycle analysis, samples were harvested, washed twice in PBS, and then fixed in ice-cold 70% ethanol at −20°C overnight. The fixed cells were treated with RNase A (R4875, Sigma-Aldrich) for 30 min at room temperature before the addition of 5 μl/ml propidium iodide (PI, P4864, Sigma-Aldrich) for 10 min in the dark and analysis by flow cytometry.

### Colony formation assay

In brief, 100 HCC cells in 4 ml medium were seeded in 6-well plate and allowed to attach overnight. The next day, the medium was replaced with fresh medium containing different concentrations of lonafarnib, and the cells were incubated at 37°C for 14 days without any disturbance. Following incubation, the medium was removed, and colonies were fixed with methanol for 15 min at room temperature prior to crystal violet (C01201, Beyotime) staining. Colony counting was performed with ImageJ software. The experiment was carried out twice in triplicate.

### Rhodamine-123 (RHO123) staining assay

Cells stably transfected with ABCB1 were seeded in 6-well plates and allowed to attach overnight. The cells were treated with various concentrations of lonafarnib for 3 h at 37°C before 5 μM RHO123 (83702, Sigma-Aldrich) was added to the cells and incubated for another 3 h in darkness at 37°C to measure the level of drug accumulation. Then, the cells were washed three times with ice-cold PBS and observed under a fluorescence microscope (Olympus IX73).

### Analysis of the synergistic effect of lonafarnib in combination with doxorubicin or sorafenib

The effect of drug combination was assessed according to the median effect principle, described by Chou et al. [42] First, we constructed dose-response curves to analyze the cytotoxic effects of lonafarnib, doxorubicin or sorafenib alone and combination of lonafarnib with doxorubicin or sorafenib in HCC cells using the CCK-8 assay. The data were used to determine the ‘combination index’ using the following equation: CI = (D)1 / (Dx)1 + (D)2 / (Dx)2, where (D)1 and (D)2 are the combination doses that kill x% of cells, and (Dx)1 and (Dx)2 are the doses of each drug alone that kill x% of cells. The combination index theorem by Chou-Talalay offers quantitative definition for additive effect (CI = 1), synergistic effect (CI < 1), and antagonistic effect (CI > 1) in drug combinations.

### Statistical analysis

SPSS software version 19.0 and GraphPad Prism 5 were used to perform the statistical analyses. The results were expressed as the mean ± standard deviation, and represent the average values from 2–3 values/experiments; experiments were repeated at least twice. The significance of variance between groups was determined by Student's *t* test. All statistical tests were two-sided, and *P* < 0.05 was considered statistically significant.

## Abbreviations

ABC: ATP-binding cassette
ABCB1: ATP-binding cassette subfamily B member 1
CCK-8: Cell Counting Kit-8
CI: combination index
DMSO: dimethyl sulfoxide
Dox: Doxorubicin
ECL: enhanced chemiluminescence
fa: affected fraction
FBS: fetal bovine serum
HCC: hepatocellular carcinoma
Lona: Lonafarnib
MAPK: mitogen activated protein kinase
MDR: multidrug resistance
MRPs: multidrug resistance-associated proteins
OS: overall survival
PVDF: polyvinylidene fluoride
qRT-PCR: real-time quantitative PCR
RHO123: rhodamine-123
RTKs: receptor tyrosine kinases
Sora: Sorafenib
